# The island rule-like patterns of plant size variation in a young land-bridge archipelago: Roles of environmental circumstance and biotic competition

**DOI:** 10.1016/j.pld.2024.12.001

**Published:** 2024-12-14

**Authors:** Zengke Zhang, Wensheng Chen, Zengyan Li, Wentao Ren, Ling Mou, Junyong Zheng, Tian Zhang, Hantang Qin, Liyi Zhou, Bile Sai, Hang Ci, Yongchuan Yang, Shekhar R. Biswas, Enrong Yan

**Affiliations:** a Zhejiang Zhoushan Island Ecosystem Observation and Research Station, and Zhejiang Tiantong Forest Ecosystem National Observation and Research Station, School of Ecological and Environmental Sciences, East China Normal University, Shanghai 200241, China; b Institute of Eco-Chongming (IEC), 3663 N. Zhongshan Rd., Shanghai 200062, China; c Key Laboratory of the Three Gorges Reservoir Region's Eco-Environment, Ministry of Education, Chongqing University, Chongqing 400030, China

**Keywords:** Insular dwarfism and gigantism, Island biogeography, The environmental stress hypothesis, The reduced herbivory hypothesis, The relaxed competition hypothesis, The resource availability hypothesis

## Abstract

The island rule, a general pattern of dwarfism in large species to gigantism in small species on islands relative to mainland, is typically seen as a macroevolutionary phenomenon. However, whether the ecological processes associated with abiotic and biotic factors generate a pattern of plant size variation similar to the island rule remains unknown. We measured plant height for 29,623 individuals of 50 common woody plant species across 43 islands in the Zhoushan Archipelago (8500 years old and yet to undergo major evolutionary adaptation) and the adjacent mainlands in China. We found pronounced variations in plant height, similar to those of the island rule. Interestingly, islands with low resource availability, such as low soil organic matter content and low precipitation, had a high degree of dwarfism; islands experiencing high environmental stress, such as high soil pH, had a high degree of dwarfism; and islands experiencing less plant–plant competition had a high degree of gigantism. The magnitude of plant dwarfism was higher on small and remote islands than on larger and nearer islands. These results highlight the importance of ecological processes associated with abiotic and biotic conditions in shaping the island rule-like patterns of plant size variation. Since our studied archipelago is too young to undergo major evolution, ecological processes likely played a prominent role in generating the observed pattern, challenging the notion that the evolutionary process is the dominant factor underlying the island rule. Future studies on the island rule need to perform experiments to disentangle evolutionary from ecological mechanisms.

## Highlights

• Young land-bridge archipelago features the island rule-like patterns of plant size variation.
• Environmental circumstances and plant competition generate a pattern of plant size variation similar to the island rule.
• The magnitude of plant dwarfism was higher on small and remote islands than on larger and nearer islands.
• Future studies on the island rule need to perform experiments to disentangle evolutionary from ecological mechanisms.

## 1 Introduction

Organism body size varies over 21 orders of magnitude (West et al., 1999) and scales with the metabolic rates of individuals, as well as population growth and mortality rates, generating profound variability in the structure and function of populations and communities (Giometto et al., 2013). Among many fascinating patterns of organism body size variation, the island rule is a classic insular pattern (Foster, 1964; Van Valen, 1973). The island rule is typically regarded as a macroevolutionary phenomenon in which small species evolve to large while large species evolve to small on islands in relation to their mainland relatives (Foster, 1964; Van Valen, 1973; Benitez-Lopez et al., 2021). Empirical support for the island rule has been found in plants (Biddick et al., 2019; Biddick and Burns, 2021) and animals (Lomolino, 1985; Benitez-Lopez et al., 2021). However, plant size variation between islands and mainlands could also result from within-species phenotypic plasticity (Arpat et al., 2009) associated with the island-to-mainland variation in abiotic and biotic factors. The island rule-like patterns of plant size variation may thus be generated by both evolutionary (Foster, 1964; Van Valen, 1973) and ecological processes (Arpat et al., 2009). Still, most research examining the island rule in plants (Biddick et al., 2019; Biddick and Burns, 2021) or animals (Lomolino, 1985; Benitez-Lopez et al., 2021) disregard ecological processes and mainly focus on evolutionary adaptation.

**Fig. 1:**
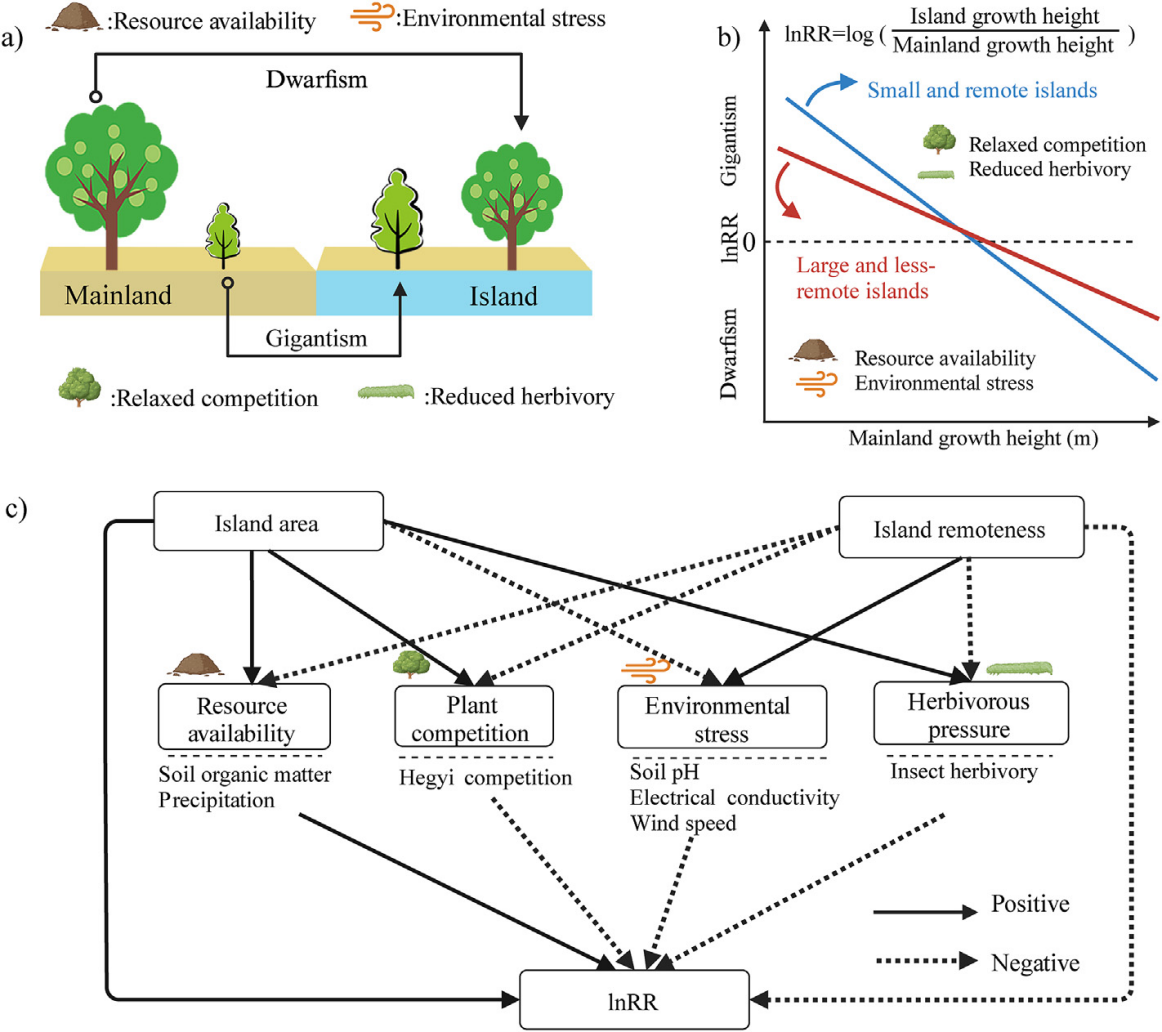
Hypothesized effects of resource availability, environmental stress, competition, and herbivory on the island rule-like patterns of plant size variation (a) and our approach to examine the island rule-like patterns (b). We aim to quantify the island rule-like patterns of plant size variation by conducting regression of the log response ratio (lnRR) of plant height between island and mainland individuals against the mainland plant height in the young insular systems. Here, the lnRR-value > 0 means gigantism, and lnRR-value < 0 means dwarfism. When the regression is significant, a positive intercept and negative slope indicate the island rule-like patterns of plant size variation (b). We argue that island area and remoteness can also directly and indirectly impact the island rule-like patterns of plant size by mediating resource availability, environmental stress, competition, and herbivory (c). We expect that compared to the large and less remote islands (red line), the regression slope between lnRR and mainland plant height will be steeper in small and remote islands (blue line) due to fewer competitions and herbivores but higher environmental stress and resource limitation (b).

An essential step towards understanding the roles of ecological processes on the island rule-like patterns is to identify the plant size variation on young islands yet to undergo major evolutionary adaptation. Without major evolutionary adaptation, plant growth form variation among young islands and mainland primarily results from phenotypic plasticity associated with abiotic and biotic drivers, including resource availability (Lomolino et al., 2012), environmental stress (Meiri et al., 2004; Roberto et al., 2023), plant–plant competition, and herbivory (Burns, 2016; Roberto et al., 2023) (Fig. 1a). This implies that island plants gigantic or dwarf relative to their mainland counterparts may be an ecological phenomenon caused by island’s abiotic and biotic contexts. For instance, islands’ unfavorable growing conditions associated with the scarcity of soil water and nutrients could dwarf island plants (Biddick and Burns, 2021). Islands’ stressful environmental conditions related to high soil salinity and strong wind could also encourage plants to invest more energy and biomass for metabolic process and mechanic support at the expense of vertical growth (Westoby et al., 2002; Marks et al., 2016), resulting in dwarfism. However, relatively low seasonality on islands may favor plant growth and survival, facilitating larger growth forms on islands relative to mainlands. Plant richness and herbivore pressure are generally low on islands. The low plant richness would likely result in low interspecific competition for limited resources, such as soil nutrients, which may promote plant growth and favor tall individuals on islands (Biddick et al., 2019). The low herbivore pressure on islands may also minimize damage to aboveground production and benefit plant growth and size (Benitez-Lopez et al., 2021; Zizka et al., 2022).

Meanwhile, island area and remoteness could impact plant size directly, and indirectly via mediating resource availability, environmental stress, plant–plant competition, and herbivory pressure (Fig. 1c). Small and remote islands are generally characterized by lower plant richness and herbivore pressure compared to large and less remote islands (Benitez-Lopez et al., 2021; Zizka et al., 2022). Plant species growing on small and remote islands may thus experience less intense interspecific competition or herbivore damage than those growing on large and less remote islands. Ecological release from competition and herbivory could allow resource-demanding and herbivore-suppressed species to flourish and become gigantic on small and remote islands (Angerbjorn, 1986). However, environmental stress and resource limitation are generally high on small and remote islands (Negoita et al., 2016; Schrader et al., 2021), restricting plant growth and favoring dwarfism (Jacquet et al., 2017). Altogether, the magnitudes of dwarfism or gigantism of island plants caused by environmental contexts and biotic interaction are likely to be higher on small and remote than on large and less remote islands (Fig. 1b).

Here, we measured plant height for 29,623 individuals of 50 common woody plant species across 43 islands in a young land-bridge island system in Zhoushan Archipelago (8500 years) and the adjacent mainland in eastern China. We mainly focus on three questions: 1) By comparing the plant height of island-mainland species pairings, we first examined whether there is a systematic pattern of plant height variation similar to the island rule. We hypothesize that if island plants’ size variation follow the patterns of the island rule in very young islands (probably leaving not enough time for major evolutionary adaptations), it will demonstrate the importance of the island’s abiotic and biotic factors underlying the potential island-to-mainland plant height variation. That would also imply that ecological processes are prominent in generating the island rule-like patterns of plant height variation rather than only evolutionary adaptation. 2) We examined whether resource availability, environmental stress, plant–plant competition and insect herbivory can explain the observed pattern of plant size variation. We anticipate that high resource limitation and environmental stress could cause dwarfism; however, low plant–plant competition and insect herbivory could result in gigantism. 3) We further examined how island area and remoteness impact the island rule-like patterns of plant size directly and indirectly through mediating resource availability, environmental stress, plant competition, and herbivorous pressure. We hypothesize that small and remote islands limit plant growth directly, and indirectly via reduced competition and herbivory to favor gigantism; alternatively, decreasing resource availability and increasing environmental stress could favor dwarfism.

## 2 Material and methods

### 2.1 Study region, geological history, climate and vegetation

This study was conducted in a mainland-island ecotone (spanned a geographical range from 29.14° to 30.85° N in latitude, 121.20°–122.90° E in longitude) spreading over a distance of about 250 km from Ninghai city in the mainland Ningbo region to Huaniao Island, located in Zhejiang province, eastern China (Fig. S1). The study region is characterized by a well-distinct climate gradient. The Zhoushan Archipelago belongs to the land-bridge islands, comprising 4696 islands and reefs. Of these, 1339 islands are larger than 500 m^2^. The largest island is Zhoushan Main Island, with an area of 502.65 km^2^ (including the tidal zone), and the highest elevation is 544.4 m on Taohua Island (Xu et al., 2023). The landscape of the mainland Ningbo region is characterized by a mixture of plains, basins, and low hills (4–900 m above sea level), with low hills being the dominant landscape element, inlaid in a fragmented pattern on the plains. Soil nutrients and water availability are generally higher on the mainlands than on islands (Fig. S2).

Geologically, the Zhoushan Archipelago and the low hills of Ningbo region belong to Tiantai Mountain’s north-eastern extension. Since the Quaternary, the Zhoushan Archipelago has been united and separated from the mainland several times due to the influence of alternated warming and cooling in climate and neotectonics movements in geography. Specifically, it was connected with the mainland during the ice age and separated from the mainland during the interglacial period. Since the late Pleistocene (25,000–18,000 years ago), the sea level has dropped 130–160 m; the Zhoushan Archipelago has been connected to the mainland again, with the climate turning cold. In the early Holocene (about 8500 years ago), the Zhoushan Archipelago was separated from the mainland due to the largest marine transgression (Zheng et al., 2006; Xu et al., 2023). The geographic history suggests insufficient time for major evolutionary adaptation of organisms in this young insular system.

The climate is typically subtropical monsoon, with hot and humid summers and warm winters. Across the mainland-island ecotone from southwestern Ninghai city to northern east Huaniao Island, the mean annual precipitation decreases from 1403–976 mm, most occurring between April and September. The mean annual temperature decreases from 16.83 to 14.34 °C. Mean annual wind velocity increases from 3.6 to 5.0 m/s. These shifted patterns of climate factors reflect a strong gradient in precipitation.

With decreasing precipitation from south to north, natural vegetation shifts from evergreen broadleaved forest, evergreen and deciduous mixed forest to deciduous broadleaved shrubland successively. The community structure of these vegetations is integral, and woody plants grow healthily, with the maximum plant height varying remarkably across and within species in this mainland-island ecotone. The Zhoushan Archipelago shared the same ancestral species pool with the mainland Ningbo region (Xu et al., 2017), thus providing sufficient island-mainland species pairs for exploring the island rule-like patterns of plant size.

### 2.2 Vegetation survey, plant species selection and growth height estimation

To investigate plant size and community structure, we chose intact and semi-intact naturally regenerated vegetation stands from the Zhoushan Archipelago (cf. islands) and the low hills of the Ningbo region (cf. mainlands). We established 110 20 m × 20 m forest plots and 84 10 m × 10 m shrubland plots on 43 islands across the Zhoushan Archipelago. The area of the studied islands ranged from 0.004 to 515.70 km^2^, and the island remoteness (i.e., the distance from the mainland Ningbo) ranged from 2.79 to 211.90 km (Xu et al., 2023). The number of plots per island ranged from 1 to 10 (mean 4.5 plots per island) and was determined based on the island's area (correlation between island area and number of plots, r = 0.78, *P* < 0.001). Following the same approach, we established 94 20 × 20 m woody vegetation plots across the low hills of mainland Ningbo. These woody vegetation plots encapsulate a wide range of community structures, species composition, plant growth forms, and environmental conditions of the Ningbo region.

For each plot, all woody plants taller than 0.5 m were tagged, identified to the level of a species, geo-referenced (position coordinates mapping), and measured for height. Island plants are generally smaller. Individuals taller than 0.5 m accounted for nearly 90% and 98% of the total richness and abundance of woody plants, respectively, suggesting that surveying woody plants taller than 0.5 m effectively capture most of the plant diversity. We used a folding rule to measure the height for smaller plants, a telescoping pole for plants up to 15 m high, and a Vertex meter (Vertex-Ⅳ, Haglof, Dalarna, Sweden) for plants higher than 15 m. We also measured the diameter at breast height (DBH, i.e., 1.3 m above the root collar) for individuals taller than 1.5 m. However, we measured the diameter at 0.45 m above the root collar for smaller plants (i.e., 0.5 m–1.5 m in height). Stem density ranged from 0.17 to 5.44 stems/m^2^ and from 0.15 to 1.54 stems/m^2^, stem basal area on a ground area basis ranged from 20 to 2748 cm^2^/m^2^ and from 39 to 2715 cm^2^/m^2^, for our sampled plots in the Zhoushan Archipelago and the mainland Ningbo, respectively.

Across 43 studied islands in the Zhoushan Archipelago, we measured 25,970 individuals from 174 species, 120 genera and 62 families. We also measured 21,902 individuals belonging to 181 species, 100 genera and 63 families in the mainland Ningbo. Across the mainland-island ecotone, 94 species, including *Camellia fraterna*, *Eurya loquaiana* and *Schima superba* (Theaceae), *Castanopsis carlesii*, *Castanopsis sclerophylla* and *Castanopsis fargesii* (Fagaceae), *Litsea elongata* (Lauraceae), *Rhododendron ovatum* (Ericaceae), appeared exclusively on the mainland. A total of 86 species, including *Eurya emarginata* (Theaceae), *Pittosporum tobira* (Pittosporaceae), *Broussonetia papyrifera* and *Morus alba* (Moraceae), *Clerodendrum trichotomum* (Lamiaceae), appeared solely on the islands. And 88 species, including *Lithocarpus glaber* and *Cyclobalanopsis glauca* (Fagaceae), *Loropetalum chinense* (Hamamelidaceae), *Symplocos sumuntia* and *Symplocos setchuensis* (Symplocaceae), *Machilus thunbergii* and *Camphora officinarum* (Lauroideae), were common in both mainland and islands. We selected 50 species common in the Zhoushan Archipelago and mainland Ningbo to examine the species-level patterns of plant size variation between islands and the mainland. The selected 50 species represent the two major growth forms, i.e., tree and shrub (Table S1).

We used the plant maximum height as a proxy for plant size to avoid potential confounding effects of varying plant age. We determined the plant maximum height by computing the 95th percentile of plant height across all individuals of a given species across the surveyed plots, separately for mainland Ningbo and each of the 43 islands. This approach does not assume invariability in the plant maximum height; instead, this approach considers the upper limit of height a species can reach on a given island. A strong correlation (Pearson’s correlation coefficient, r = 0.85, *P* < 0.001) between the 95th percentile of plant height and species basal diameter across species indirectly confirmed the reliability of our approach (Fig. S3).

### 2.3 Quantification of island environment conditions

To characterize the effects of island resource availability and environmental stress on plant size, we measured plot-level soil organic matter content, pH, and electrical conductivity and gathered precipitation and wind speed data. Using a metal corer, we collected four soil samples from the 0–20 cm soil layer after removing the litter layer at four quadrant centers within each plot (*N* = 194 plots). Four soil samples taken from a plot were mixed to make a composite sample for the plot. Soil samples were placed in a sealed plastic bag and immediately shifted to the laboratory. We air-dried those samples for 30 days, grounded them with a mortar and pestle, and passed them through a 2 mm sieve. Air-dried soil samples were then analyzed for soil organic matter content using the H_2_SO_4__K_2_CrO_7_ oxidation method. At the same time, soil pH and electrical conductivity were measured using a pH electrode (PHSJ-4F, INESA, China) and a soil conductometer (DDS-SO7A, INESA, China).

We extracted plot-specific data on precipitation in the warmest quarter and wind speed for 194 plots from the WorldClim v.2.0 (http://worldclim.org) (Fick and Hijmans, 2017). Data extraction and analyses were conducted using Extract Multi Values to Points in ArcGIS 10.1.

In the subsequent statistical analysis, the values of soil properties and climate factors were averaged for all plots per island to obtain island-level values.

### 2.4 Quantification of plant–plant competition

To determine the effect of plant–plant competition on plant size, we calculated the Hegyi competition index ($H_{ij}$, equation (1)), which indicates the competitive pressure of all individuals in a plot. Because the Hegyi competition index is a distance-dependent index, it is usually strongly correlated with plant size (Contreras et al., 2011).

(1) $$H_{ijk}=\sum_{j=1}^{n}\frac{{DBH}_{jk}}{{DBH}_{ik}}*\frac{1}{{\left({Dist}_{ij}\right)}_{k}}$$

Where, $H_{ijk}$ is the Hegyi competition index for the *i*th individual in the *k*th plot; ${DBH}_{jk}$, ${DBH}_{ik}$ and ${Dist}_{ijk}$ are the DBH of the competing tree *j*, the DBH of the target tree *i* and the distance between the competing individual and the target individual, respectively; and *n* is the total number of competing individuals of the target individual *i*. To correct for edge effects, we used an area-weighted edge correct Hegyi competition (Luo et al., 2019).

Because species under the lowest competitive pressure can achieve their maximum height in situ, we used a given species’ lowest competitive index value in a given plot to indicate the lowest competitive pressure. The lowest competitive values were calculated for the 5th percentile of $H_{ijk}$ for each species at the island level (i.e., combined all plots per island). The strong correlation (r = 0.62, *P* < 0.001, Fig. S4) between the maximum plant height and 5th percentile of $H_{ijk}$ confirmed that the lowest competitive index was a reliable proxy of the effect of plant competition on plant size.

### 2.5 Quantification of insect herbivorous pressure

Herbivorous insects, including *Lebeda nobilis*, *Trabala vishnou*, *Trabala pallida*, *Dendrolimus punctata* (Lasiocampidae), *Pidorus atratus* (Zygaenidae), *Papilio bianor* (Papilionidae), are the primary consumers of woody plant leaves in the Zhoushan Archipelagos. We measured the proportion of plant leaf damage by insects to assess the herbivore pressure on plant size. For that, we focused on the common plant species and selected three well-grown and healthy adult individuals for each species in each plot (*N =* 194 plots). For each individual, four branches (≥ 20 leaves) were randomly collected from mid-canopy around the east, west, south, and north directions using a long-arm arm tree pruner. Four collected branches per individual were placed in a large plastic bag and then immediately brought back to the laboratory. In the laboratory, we carefully counted the number of leaves damaged by insect herbivory and the number of leaves attached to a branch. We calculated the ratio of the number of insect-damaged leaves to the total number of leaves on a branch to represent insect herbivorous pressure. The mean herbivore damage of four branches across three individuals was calculated to represent species-level insect herbivorous pressure. We calculated the community-weighted mean (CWM, equations (2), (3))) value to represent island-level insect herbivorous pressure.

(2) $$CWM=\sum_{i=1}^{s}p_{i}h_{i}$$

(3) $$p_{i}=\frac{\text{Relative}\;\text{abundance}+R\text{elative}\;\text{dominance}}{2}*100\%$$

Where CWM is the community-weighted mean value for insect herbivorous pressure at the island level, *s* denotes the number of species on the island, *p_i_* is the relative important value of species *i* and *h_i_* is the insect herbivory pressure of species *i*. The relative abundance of each species was assessed based on the stem number of that species divided by the total stem number of all species. Relative dominance was calculated based on the basal area of each species divided by the total basal area of all species.

### 2.6 Data analyses

To test the island rule-like patterns of plant size variation, we first calculated the log-transformed response ratio (lnRR) of the maximum height of a species from a given island to the maximum height of that species on the mainland (equation (4)):

(4) $$\text{lnRR}=\ln\;\left(\frac{{H\_\max}_{i}}{{H\_\max}_{m}}\right)$$

Where, ${H\_\max}_{i}$ represents the maximum height of species from a given island and ${H\_\max}_{m}$ represents the maximum height of that species from the mainland. Response ratios larger than zero indicate gigantism, whereas response ratios lesser than zero indicate dwarfism.

Next, we used linear mixed effect models to regress lnRR against the maximum plant height of the mainland counterpart species with populations on each island and the mainland by totally involving 690 island-mainland species pairings (sum of population pairs between individual island and mainland across 50 species). Because plant growth form (tree and shrub) and species taxa can obscure the island rule-like patterns (Biddick et al., 2019; Benitez-Lopez et al., 2021), we included them as random factors in our model. Including these factors as a random effect could also account for the potential confounding effects associated with plant phylogeny. To test the robustness of the island rule-like pattern, we first averaged the maximum plant height for each species (*N* = 50 species) occurring in all islands to derive the species-specific average plant height on islands. We calculated the maximum plant height for each species (*N* = 50 species) occurring in the mainland (i.e., plots in the low hills of mainland Ningbo) to derive the species-specific plant height on the mainland. We then regressed the species-specific average plant height on islands against the species-specific plant height on the mainland. The significant negative relationship would indicate that differing growth forms of island plants follow the patterns of the island rule.

To evaluate the effects of abiotic and biotic drivers on the island rule-like patterns of plant size, we modeled interactions between mainland plant size and each explanatory variable (equation (5)).

(5) $$\text{lnRR}\;∼\beta_{0}+\beta_{1}{H\_\max}_{m}+\beta_{2}D+\beta_{3}\left({H\_\max}_{m}\times D\right)+{\;\pi }_{\text{GF}}+\pi_{\text{SI}}+\varepsilon$$

Where, $\beta_{i}$ are partial regression coefficients to be estimated. *D* represents the explanatory variables relating to each of the hypotheses of resource availability, environmental stress, relaxed competition, and reduced herbivory hypothesis. ${\;\pi }_{\text{GF}}$ and $\pi_{\text{SI}}$ are random effect factors of “growth form” and “species taxa”, and ε represents sampling errors.

We included soil organic matter content (i.e., soil nutrient resource) and the precipitation of the warmest quarter (i.e., water supply) as a proxy for resource availability on islands (Zizka et al., 2022). Soil pH and electrical conductivity (i.e., soil salt stress), and wind speed (i.e., wind stress) were used as proxy for environmental stress on islands. The Hegyi competition index was employed to measure competition pressure on islands. We included insect herbivore damage intensity as a proxy for herbivory pressure on islands. In addition, island area and remoteness were included in the model separately, as these island biogeographic properties indirectly impact the island rule-like patterns of plant size through mediating intensity of resource availability, environmental stress, relaxed competition, and reduced herbivory. Most correlations among these explanatory factors were statistically low, except for the correlation between island remoteness and precipitation (Fig. S5). To control overlapping explanations among these drivers, we individually modeled plant size against these variables (Benitez-Lopez et al., 2021).

The effects of these drivers on the island rule-like patterns of plant size might depend on growth forms, i.e., small, medium, and large species. We thus modeled interactions between mainland plant size and each of the drivers separately concerning plant size. The Quantile Function was employed to produce three samples corresponding to each driver's 10%, 50%, and 90% quantile (low-middle-high), to justify how these ecological drivers altered the relationship between lnRR and mainland plant size. If the interaction between mainland plant size and a given driver were statistically significant, it would indicate a significant variation in the regression slopes among three groups (i.e., 10%, 50%, and 90%). We used the Visreg Function to visualize regression models.

Finally, to mechanistically understand how island area and remoteness, directly and indirectly, influence the island rule-like patterns of plant size via mediating these ecological drivers, we employed piecewise structural equation modeling (SEM). The SEM was built based on our priori hypotheses (Fig. 1c). First, we selected explanatory variables that had significant impacts on the island rule-like patterns of plant size based on the above linear mixed effect models and excluded variables with high correlation (|r| ≥ 0.7). Then, we built an SEM by focusing on whether island area and remoteness directly and indirectly impact lnRR by mediating these ecological drivers. However, this model was not a good fit based on Fisher’s C statistic test (*P* < 0.05). We, therefore, removed the non-significant variables to improve the overall performance of the SEM models. After that, we compared the model AIC values (Akaike information criterion value) and selected the final model based on the lowest AIC value. We fitted SEM by accounting for the random effects of species taxa and growth form using the ‘psem’ function of piecewiseSEM R-package (Schrader et al., 2020).

Before the analyses, we checked all the explanatory variables and transformed them into log_10_ to meet normality assumptions. These transformed variables were standardized (mean = 0, SD = 1) to facilitate comparison of the coefficient estimates. All analyses were performed in R v.4.2.3 using the packages *lme4*, *visreg* and *piecewiseSEM*.

## 3 Results

### 3.1 The island rule-like patterns of plant size

**Fig. 2:**
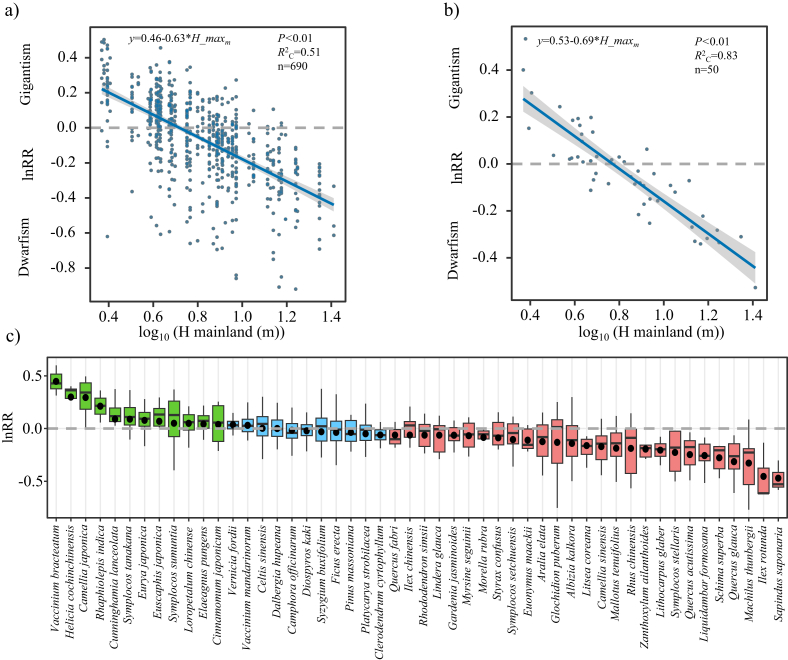
The relationship between lnRR (log response ratio of island plant size against mainland plant size) and mainland plant size with 690 island-mainland species pairings based on plant height at individual island (a) and 50 island-mainland species pairings based on plant height averaged across islands (b). Panel (c) showed a graded trend from gigantism (green) to dwarfism (red). However, there was no change in some species (blue) on islands relative to their mainland counterpart.

The log-response ratio of the island to mainland plant height (lnRR) was significantly negatively associated with the mainland plant height (Fig. 2a,b), suggesting a general pattern of dwarfism in large species to gigantism in small species on islands relative to the mainland. When we examined the pattern for individual species (*N* = 50 species), we found that 27 species tended towards dwarfism (cf. reduction in height), 12 species tended towards gigantism (cf. increase in height), and 11 species, including *Camphora* *officinarum*, *Syzygium buxifolium*, and *Dalbergia hupeana,* remained stable (Fig. 2c).

### 3.2 Response of the plant size patterns to ecological and biogeographic drivers

**Fig. 3:**
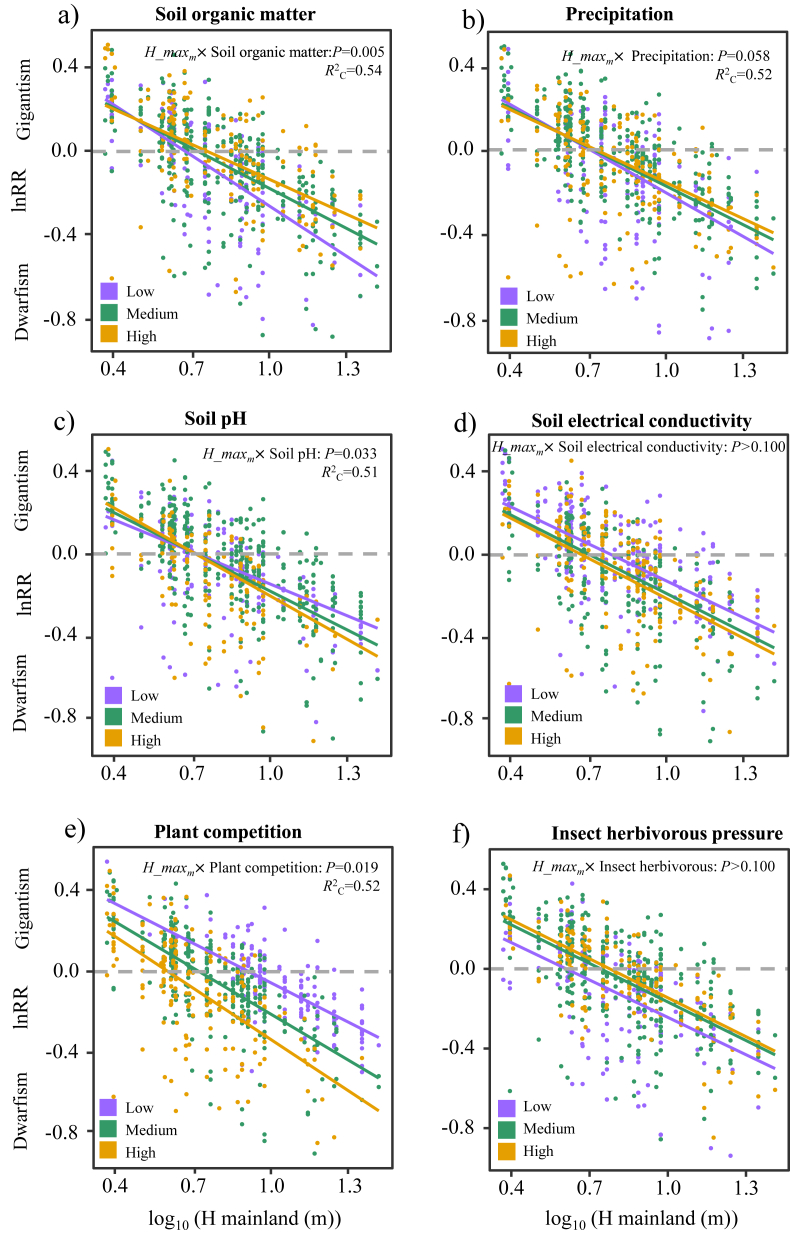
The effects of soil organic matter content (a), precipitation (b), soil pH (c), soil electrical conductivity (d), plant competition (e), and insect herbivorous pressure (f) on the island rule-like patterns of plant size. Ecological drivers are represented at the 10% (low), 50% (medium) and 90% (high) quantile for each variable.

Concerning the resource availability hypothesis, soil organic matter content and precipitation significantly affected the island rule-like patterns of plant size variation (Table 1; Fig. 3a,b), suggesting that low soil organic matter content and precipitation led to a higher degree of dwarfism. Concerning the environmental stress, the insular patterns of plant size variation were mostly explained by soil pH, with high soil pH (i.e., high soil salt) resulting in more pronounced dwarfism (Fig. 3c; Table 1). However, soil electrical conductivity (Fig. 3d) and wind speed did not affect the insular patterns of plant size variation (Fig. S6). Consistent with the relaxed competition hypothesis, plant size consistently became gigantism under low plant competition pressure and became dwarfism under high competition pressure (Fig. 3e; Table 1). Contrary to the reduced herbivory hypothesis, insect herbivorous pressure did not affect the insular patterns of plant size (Fig. 3f; Table 1).

**Table 1:** Parameter estimates for the linear mixed effect models testing different drivers of the island rule-like patterns of plant size.

Interaction effect	Estimate	df	t value	*P* value	*R*_C_^ 2^	*R*_m_^ 2^
${H\_\max}_{m}$ × Soil organic matter	0.10	659.08	2.82	**0.01**	0.54	0.33
${H\_\max}_{m}$ × Precipitation	0.05	673.43	1.78	**0.06**	0.52	0.31
${H\_\max}_{m}$ × Soil PH	−0.07	676.6	−2.13	**0.03**	0.51	0.31
${H\_\max}_{m}$ × Soil electrical conductivity	−0.02	663.85	−0.63	0.53	0.52	0.31
${H\_\max}_{m}$ × Wind speed	0.00	670.44	−0.13	0.89	0.51	0.30
${H\_\max}_{m}$ × Plant competition	−0.08	577.39	−2.35	**0.02**	0.52	0.40
${H\_\max}_{m}$ × Herbivorous pressure	0.03	663.37	−0.78	0.43	0.50	0.32
${H\_\max}_{m}$ × Island area	0.07	679.34	2.01	**0.04**	0.55	0.35
${H\_\max}_{m}$ × Island remoteness	−0.06	673.26	−2.00	**0.05**	0.52	0.31

Note: *R*_C_^2^ and *R*_m_^2^ represent conditional *R*^2^ and marginal *R*^2^, respectively.

**Fig. 4:**
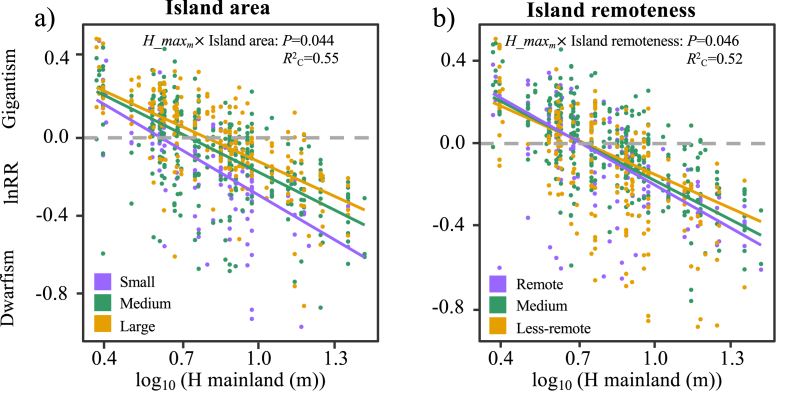
The effects of island area (a) and remoteness (b) on the island rule-like patterns of plant size. Continuous variables are represented at the 10%, 50% and 90% quantile for each extreme (small, medium, large, or remote, medium, less-remote).

Island area and remoteness significantly affected the island rule-like patterns of plant size. On average, the more steep slopes and a large proportion of the negative intercepts (lnRR < 0) in the regression relationships demonstrated a pronounced dwarfism trend on small and remote islands (Fig. 4a,b).

### 3.3 Direct and indirect effects of island area and remoteness on the insular patterns of plant size

**Fig. 5:**
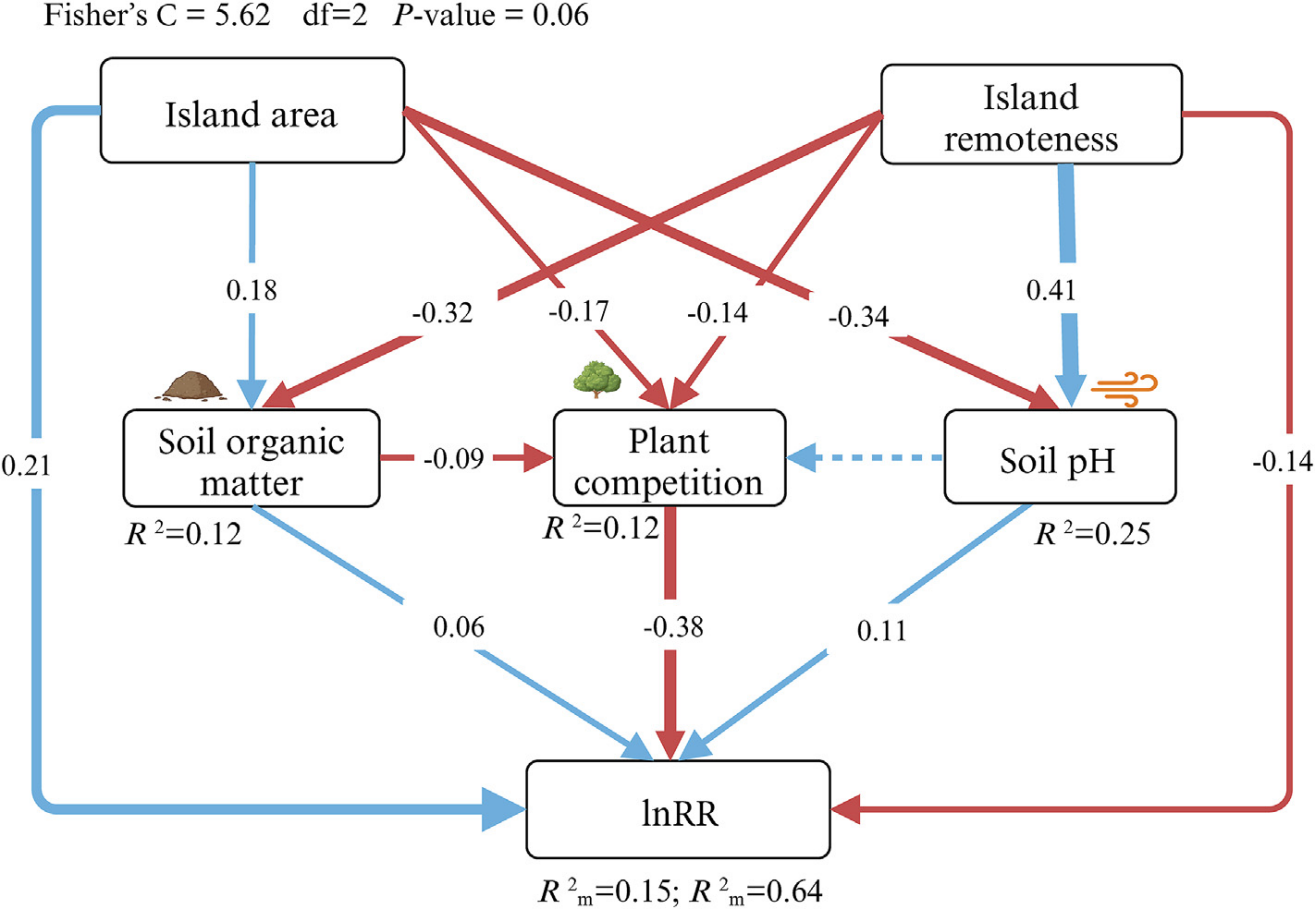
Effects of island area, island remoteness, soil organic matter content, soil pH, and plant competition on insular patterns of plant size variation. For each path, the standardized regression coefficient is shown. Solid blue and red lines represent significantly positive and negative paths, respectively, while dashed lines represent insignificant paths. Marginal (*R*^2^_m_; based on fixed effects) and conditional *R*^2^ (*R*^2^_c_; based on both fixed and random effects) are reported.

The SEM model yielded a good fit to the data (Fisher’s C = 5.62, df = 2, *p* = 0.06), and accounted for 64% of the variation in insular patterns of plant size (Fig. 5, Table S2). The island area had a significantly positive total effect on lnRR (standardized coefficient, r = 0.25) via its direct effect (r = 0.21) and indirect effects via soil organic matter content (r = 0.01), soil pH (r = −0.04), and plant competition (r = 0.06). Island remoteness had a slightly significant negative total effects on lnRR (r = −0.06) via its direct effect (r = −0.14) and an indirect effect via soil organic matter content (r = −0.02), soil pH (r = 0.05), and plant competition (r = 0.05). The island area positively affected soil organic matter content (r = 0.18) but negatively affected soil pH (r = −0.34). The island area had a significantly negative total effect on plant competition (r = −0.19) via its direct effect (r = −0.17) and indirect effect via soil organic matter content (r = −0.02). Island remoteness positively affected soil pH (r = 0.41) but affected soil organic matter content negatively (r = −0.32). Island remoteness had a significantly negative total effect on plant competition (r = −0.11) via its direct effect (r = −0.14) and indirect effect via soil organic matter content (r = 0.03). The lnRR was positively affected by soil organic matter content (r = 0.06) and soil pH (r = 0.11), while negatively affected by plant competition (r = −0.38).

## 4 Discussion

In a relatively young land-bridge archipelago yet to undergo major evolutionary adaptation, we found a striking pattern of plant height variation that closely matches the island rule. In theory, the observed pattern could result from a combination of ecological and evolutionary processes. However, given the age of the archipelago (too young to undergo major evolution), it is likely that ecological processes played a prominent role in generating the observed pattern. We also show that small and remote islands had a higher degree of dwarfism in plant size and that island area and remoteness could influence the island rule-like patterns of plant size directly and indirectly through its effects on resource availability, environmental stress, and plant competition. These results collectively imply that the variation in plant size, similar to the island rule, could be an ecological phenomenon attributed to abiotic and biotic drivers.

### 4.1 Plant size variation similar to the patterns of the island rule in very young islands

The island rule has been widely recognized as a macroevolutionary phenomenon in long-lasting isolated oceanic islands (Foster, 1964; Van Valen, 1973; Lomolino, 1985). The rule has been applied to explain body size variation for diversified organisms, including birds (Covas, 2012; Ponti et al., 2023), mammals (Lomolino, 2005), reptiles (Novosolov et al., 2013; Benitez-Lopez et al., 2021), and plants (Burns, 2016; Biddick et al., 2019). However, the island rule remains debated (Lokatis and Jeschke, 2018): some studies find confirmatory evidence (Lomolino, 1985; Clegg and Owens, 2002; Welch, 2009; Lomolino et al., 2012), and others do not (Meiri et al., 2004, 2008; Itescu et al., 2014, 2018). Contradictory results among studies imply that ecological processes beyond evolutionary adaptation might play essential roles in shaping plant size variation among islands and the mainland (Li et al., 2021). For instance, although both ecological and evolutionary processes can generate a similar pattern of organism body size variation (Ryan and Yoder, 1997), studies examining the island rule disregard the potential roles of ecological processes.

We addressed the issue by comparing plant height variation between young islands (8500 years) and the mainland, assuming that young islands (on an evolutionary time scale) are yet to undergo major evolutionary adaptation. We expected that if the young island plant height variation follows a similar pattern to the island rule, it will showcase the importance of the island's abiotic and biotic factors underlying the island-to-mainland plant height variation. Indeed, our analyses revealed a broad pattern of dwarfism in large species to gigantism in small species on young islands relative to their mainland counterparts, suggesting that, instead of macroevolutionary processes alone, ecological processes also played a prominent role in driving the island rule-like patterns of plant size variation. Li et al. (2021) reported a similar pattern from the young land-bridge islands of Thousand Island Lake, where mammal species’ body size variation pattern was similar to the island rule. Although the genus and family level analyses make little sense for the classical island rule (evolution after island colonisation), the island rule-like patterns of plant size variation were also pronounced at genus and family levels (Fig. S7a,b). Since the formation of genera and families is much older than any island colonisations, the systematic patterns we observed here would confirm that differing growth forms between the island and mainland taxa could result from an ecological phenomenon caused by environmental conditions and biotic interaction; it is less likely that evolutionary adaptations generated the pattern observed here. Altogether, it is likely that ecological processes that shape within-species plasticity played an important role in the island rule-like patterns of plant size in our studied young land-bridge archipelagos.

Although our study evidenced the roles of abiotic and biotic factors in shaping the island rule-like patterns for plant size variation in a young insular system, these results may also apply to old insular systems. This is because environmental conditions on old islands are often harsh, which can impact plant growth forms. For instance, studies examining the island rule in the old insular systems have inferred processes from patterns without providing evidence of evolutionary adaptation, such as changes in the genes (Lomolino et al., 2012; Cox and Burns, 2017; Biddick et al., 2019; Ponti et al., 2023). Putting our results into the context of old insular systems would imply that, beyond evolutionary adaptation, island abiotic and biotic conditions can also generate phenotypic variation in plant growth forms and a pattern of plant size variation identical to the island rule. Since ecological and evolutionary effects on insular patterns of organism body size are intimately intertwined (Arpat et al., 2009), future in-depth studies are crucially required to disentangle the confounding effects of ecological and evolutionary drivers on the island rule.

It is worth considering that founder effects or micro-evolutionary changes (which can occur within a few generations) likely occur in our studied young archipelagos, which could induce genetic differences between island and mainland populations of plants. Such genetic changes may cause plant size variation, thus potentially generating the island rule-like patterns of plant growth forms (Meiri et al., 2008; Biddick and Burns, 2021; Roberto et al., 2023). In this study, we acknowledged that we cannot fully discount the effects of such evolutionary drift on the island rule-like patterns of plant size variation. In the next, conducting a transplanting or a common garden experiment to differentiate plastic and genetic effects and utilizing molecular methodologies to discern evolutionary effects between mainland-island population pairs would be an exciting avenue to proceed (Burns et al., 2012; Li et al., 2021).

### 4.2 How environmental conditions and biotic interaction generate the island rule-like patterns of plant size

We anticipate that a high level of resource limitation and environmental stress could cause dwarfism, and a low level of plant–plant competition and insect herbivory could result in gigantism. Consistent with our hypothesis, we found that resource availability, environmental stress and relaxed competition jointly drive the convergence of plant size on islands. This result suggests that plant size convergence on islands may result from environmental conditions and biotic interaction (Meiri et al., 2008; Biddick and Burns, 2021; Roberto et al., 2023). In line with the resource availability hypothesis, we observed that tall plant species exhibited a high degree of dwarfism on unfertile (low soil organic matter content) and dry (low precipitation) islands (Fig. 3a,b). Low soil nutrients and water supply might limit island plant growth. In our studied systems, soil organic matter content and precipitation in the warmest quarter varied along the mainland-to-island gradient from 68.57 g/kg to 46.04 g/kg and from 432 mm to 387 mm, respectively (Fig. S2). Decreasing resource availability on the islands is undoubtedly disadvantageous to island plant growth, promoting dwarfism.

Soil pH is another important driver of plant size (Moles et al., 2009). Consistent with the environmental stress hypothesis, we found that high soil pH facilitated dwarfism on islands. Island soils are generally more alkaline than the mainland soil. Plants growing in alkaline island may invest more energy to resistant salt at the expense of growth, thus decreasing the growth rate on islands (Marks et al., 2016).

Consistent with the relaxed competition hypothesis, we found that low plant competition promoted gigantism. While weak competition for soil water and nutrients is conducive to increasing plant height (Luo et al., 2019), interspecific competition for light could also favor larger growth forms (Carlquist, 1974), as Darwin claimed in his wrong explanation for island woodiness (Zizka et al., 2022).

Insect herbivory did not affect the mainland-island patterns of plant size in our study. Many insects can fly long distances (Burns, 2014; Moreira et al., 2021), and our studied islands are not too far from the mainland to have a differential pattern of herbivory. It is worth considering that past empirical studies validating the reduced herbivory hypothesis focused on browsing pressure by ungulates (e.g., sheep, cattle, and deer) (Burns, 2014; Moreira et al., 2021; Moreira and Abdala-Roberts, 2024). In our studied system, deer herbivory occurs only on a few islands with better natural protection, and deer herbivory is absent on most islands due to human activities. It would be worthwhile to examine the specific influences of this herbivore on the island rule-like patterns of plant size in the future.

### 4.3 How island area and remoteness impact the island rule-like patterns of plant size

Beyond abiotic and biotic drivers, we predict that small and remote islands would limit plant growth directly and indirectly via reduced competition and herbivory to favor gigantism; alternatively, decreasing resource availability and increasing environmental stress could favor dwarfism. In general, we found that small and remote islands tended to promote dwarfism (Fig. 4a, b). The direct effect of island area and remoteness can be explained by selective extinction. As our studied system is the land-bridge islands, species on islands will likely be gradually lost over time (Si et al., 2016). For instance, larger plants with greater spatial and resource requirements are more prone to local extinction on small islands. Moreover, partially consistent with our hypothesis, small islands indirectly limit plant size via increasing plant competition and soil pH, decreasing soil organic matter content (Fig. 5 and Table S2). Small islands are generally poor in soil nutrients (Wardle et al., 2003) and receive salt from sea spray due to the small landmass buffer; these adverse factors might cause dwarfism on small islands. Our results of high plant competition on small islands contradict the ecological release hypothesis demonstrated on the oceanic islands (Benitez-Lopez et al., 2021; Biddick and Burns, 2021). It is likely that human activities heavily influenced our studied land-bridge archipelago. The loss rates of large trees and the ensuing increase rate of small individuals were greater on small than large islands (Xu et al., 2023). Therefore, poor soil nutrients and limited space per unit area for densely growing plants jointly lead to intensive competition on small islands.

Partially consistent with our hypothesis, remote islands appear to support tall plants indirectly, via reducing plant competition and increasing soil pH, and limiting plant growth via decreasing soil nutrients (Fig. 5). Decreased plant competition on remote islands may be attributable to the ecological release from resource competition pressure due to low species diversity (McNab, 2010; Si et al., 2016). In addition, remote islands are often barren and poor in soil nutrients due to little input of organic matter from sparse vegetation and subjected to more inputs of seawater from sea spray and greater salt additions, thereby increasing soil salinity (Vitousek, 2004).

## 5 Conclusion

This study provided compelling evidence that the island rule cannot simply be regarded as an evolutionary phenomenon, since ecological processes also play a significant role in shaping island rule-like patterns. Unless the individual and joint effects of ecological and evolutionary processes are distinguished, the island rule could be considered as an artifact of comparing mainland-island population pairings. We suggest that future studies on the island rule (evolution after island colonisation) must perform experiments to verify their results by controlling ecological effects.

## CRediT authorship contribution statement

**Zengke Zhang:** Writing – review & editing, Writing – original draft, Visualization, Validation, Methodology, Investigation, Formal analysis, Data curation. **Wensheng Chen:** Writing – review & editing, Data curation. **Zengyan Li:** Writing – original draft, Investigation. **Wentao Ren:** Investigation, Data curation. **Ling Mou:** Writing – review & editing, Data curation. **Junyong Zheng:** Writing – review & editing, Investigation. **Tian Zhang:** Writing – review & editing, Data curation. **Hantang Qin:** Writing – review & editing, Investigation. **Liyi Zhou:** Formal analysis, Data curation. **Bile Sai:** Investigation, Data curation. **Hang Ci:** Writing – review & editing, Investigation. **Yongchuan Yang:** Writing – review & editing, Methodology. **Shekhar R. Biswas:** Writing – review & editing. **Enrong Yan:** Writing – review & editing, Writing – original draft, Supervision, Resources, Funding acquisition, Conceptualization.

## Declaration of competing interest

The authors declare that they have no known competing financial interests or personal relationships that could have appeared to influence the work reported in this paper.
