# Supplementary material for: The island rule-like patterns of plant size variation in a young land-bridge archipelago: Roles of environmental circumstance and biotic competition
Source: Plant Divers. 2024 Dec 14;47(2):300–10. doi: 10.1016/j.pld.2024.12.001 (PMC11963187; doi:10.1016/j.pld.2024.12.001)
Supplement: Multimedia component 1 [file mmc1.docx]

**Appendix A. Supplementary Data for**

**The island rule-like patterns of plant size variation in a young land-bridge archipelago: roles of** **environmental** **circumstance and** **biotic competition**

Zengke Zhang, Wensheng Chen, Zengyan Li, Wentao Ren, Ling Mou, Junyong Zheng, Tian Zhang, Hantang Qin, Liyi Zhou, Bile Sai, Hang Ci, Yongchuan Yang, Shekhar R Biswas, Enrong Yan

**Table S1. Studied 50 species for testing the island rule-like patterns of plant size.**

| Species | Family | Genus | Growth form | The number of individuals on islands | The number of individuals on mainland |
| --- | --- | --- | --- | --- | --- |
| *Albizia kalkora* | Fabaceae | Albizia | Tree | 600 | 44 |
| *Aralia chinensis* | Araliaceae | Aralia | Shurb | 11 | 7 |
| *Camellia japonica* | Theaceae | Camellia | Shurb | 55 | 5 |
| *Camellia sinensis* | Theaceae | Camellia | Shurb | 221 | 24 |
| *Camphora officinarum* | Lauraceae | Cinnamomum | Tree | 467 | 43 |
| *Celtis sinensis* | Ulmaceae | Celtis | Tree | 565 | 39 |
| *Cinnamomum japonicum* | Lauraceae | Cinnamomum | Tree | 35 | 44 |
| *Clerodendrum cyrtophyllum* | Verbenaceae | Clerodendrum | Shurb | 16 | 125 |
| *Cunninghamia lanceolata* | Cupressaceae | Cunninghamia | Tree | 372 | 81 |
| *Dalbergia hupeana* | Leguminosae | Dalbergia | Tree | 1134 | 93 |
| *Diospyros kaki* | Ebenaceae | Diospyros | Tree | 132 | 60 |
| *Elaeagnus pungens* | Elaeagnaceae | Elaeagnus | Shurb | 334 | 4 |
| *Euonymus maackii* | Celastraceae | Euonymus | Tree | 15 | 5 |
| *Eurya japonica* | Theaceae | Eurya | Shurb | 2969 | 6 |
| *Euscaphis japonica* | Staphyleaceae | Euscaphis | Shurb | 72 | 11 |
| *Ficus erecta* | Moraceae | Ficus | Tree | 1760 | 31 |
| *Gardenia jasminoides* | Rubiaceae | Gardenia | Shurb | 387 | 15 |
| *Glochidion puberum* | Euphorbiaceae | Glochidion | Shurb | 272 | 19 |
| *Helicia cochinchinensis* | Proteaceae | Helicia | Tree | 7 | 18 |
| *Ilex chinensis* | Aquifoliaceae | Ilex | Tree | 343 | 210 |
| *Ilex rotunda* | Aquifoliaceae | Ilex | Shurb | 19 | 20 |
| *Lindera glauca* | Lauraceae | Lindera | Shurb | 82 | 72 |
| *Liquidambar formosana* | Hamamelidaceae | Liquidambar | Tree | 486 | 171 |
| *Lithocarpus glaber* | Fagaceae | Lithocarpus | Tree | 84 | 776 |
| *Litsea coreana* | Lauraceae | Litsea | Tree | 93 | 42 |
| *Loropetalum chinense* | Hamamelidaceae | Loropetalum | Shurb | 2707 | 850 |
| *Machilus thunbergii* | Lauroideae | Machilus | Tree | 572 | 360 |
| *Mallotus tenuifolius* | Euphorbiaceae | Mallotus | Tree | 1493 | 19 |
| *Morella rubra* | Myricaceae | Myrica | Tree | 35 | 138 |
| *Myrsine seguinii* | Myrsinaceae | Rapanea | Shurb | 24 | 6 |
| *Pinus massoniana* | Pinaceae | Pinus | Tree | 95 | 415 |
| *Platycarya strobilacea* | Juglandaceae | Platycarya | Tree | 561 | 20 |
| *Quercus acutissima* | Fagaceae | Quercus | Tree | 328 | 38 |
| *Quercus fabri* | Fagaceae | Quercus | Tree | 1357 | 87 |
| *Quercus glauca* | Fagaceae | Cyclobalanopsis | Tree | 529 | 532 |
| *Rhaphiolepis indica* | Rosaceae | Rhaphiolepis | Shurb | 14 | 106 |
| *Rhododendron simsii* | Ericaceae | Rhododendron | Shurb | 743 | 188 |
| *Rhus chinensis* | Anacardiaceae | Rhus | Shurb | 57 | 23 |
| *Sapindus saponaria* | Sapindaceae | Sapindus | Tree | 10 | 9 |
| *Schima superba* | Theaceae | Schima | Tree | 113 | 1136 |
| *Styrax confusus* | Styracaceae | Styrax | Shurb | 260 | 200 |
| *Symplocos setchuensis* | Symplocaceae | Symplocos | Shurb | 536 | 271 |
| *Symplocos stellaris* | Symplocaceae | Symplocos | Shurb | 54 | 197 |
| *Symplocos sumuntia* | Symplocaceae | Symplocos | Shurb | 211 | 718 |
| *Symplocos tanakana* | Symplocaceae | Symplocos | Shurb | 350 | 16 |
| *Syzygium buxifolium* | Myrtaceae | Syzygium | Shurb | 345 | 910 |
| *Vaccinium bracteatum* | Ericaceae | Vaccinium | Shurb | 75 | 5 |
| *Vaccinium mandarinorum* | Ericaceae | Vaccinium | Shurb | 130 | 105 |
| *Vernicia fordii* | Euphorbiaceae | Vernicia | Tree | 14 | 54 |
| *Zanthoxylum ailanthoides* | Rutaceae | Zanthoxylum | Tree | 108 | 3 |

**Table S2.** **The direct, indirect and total standardized effects of island area, remoteness, soil organic matter content, soil pH, and plant competition on lnRR based on the structural equation model.**

| Variables | Predictor | Pathway | Effect |
| --- | --- | --- | --- |
| lnRR | Island area | Direct effect | 0.21 |
|  |  | Indirect effect via soil organic matter | 0.01 |
|  |  | Indirect effect via soil pH | -0.04 |
|  |  | Indirect effect via plant competition | 0.06 |
|  |  | Total effect | 0.25 |
|  | Island remoteness | Direct effect | -0.14 |
|  |  | Indirect effect via soil organic matter | -0.02 |
|  |  | Indirect effect via soil pH | 0.05 |
|  |  | Indirect effect via plant competition | 0.05 |
|  |  | Total effect | -0.06 |
|  | Plant competition | Direct effect | -0.38 |
|  | Soil organic matter | Direct effect | 0.06 |
|  |  | Indirect effect via plant competition | 0.07 |
|  |  | Total effect | 0.13 |
|  | Soil pH | Direct effect | 0.11 |
| Soil organic matter | Island area | Direct effect | 0.18 |
|  | Island remoteness | Direct effect | -0.32 |
|  |  | Total effect | -0.14 |
| Soil pH | Island area | Direct effect | -0.34 |
|  | Island remoteness | Direct effect | 0.41 |
|  |  | Total effect | 0.07 |
| Plant competition | Island area | Direct effect | -0.17 |
|  |  | Indirect effect via soil organic matter | -0.02 |
|  |  | Total effect | -0.19 |
|  | Island remoteness | Direct effect | -0.14 |
|  |  | Indirect effect via soil organic matter | 0.03 |
|  |  | Total effect | -0.11 |

**
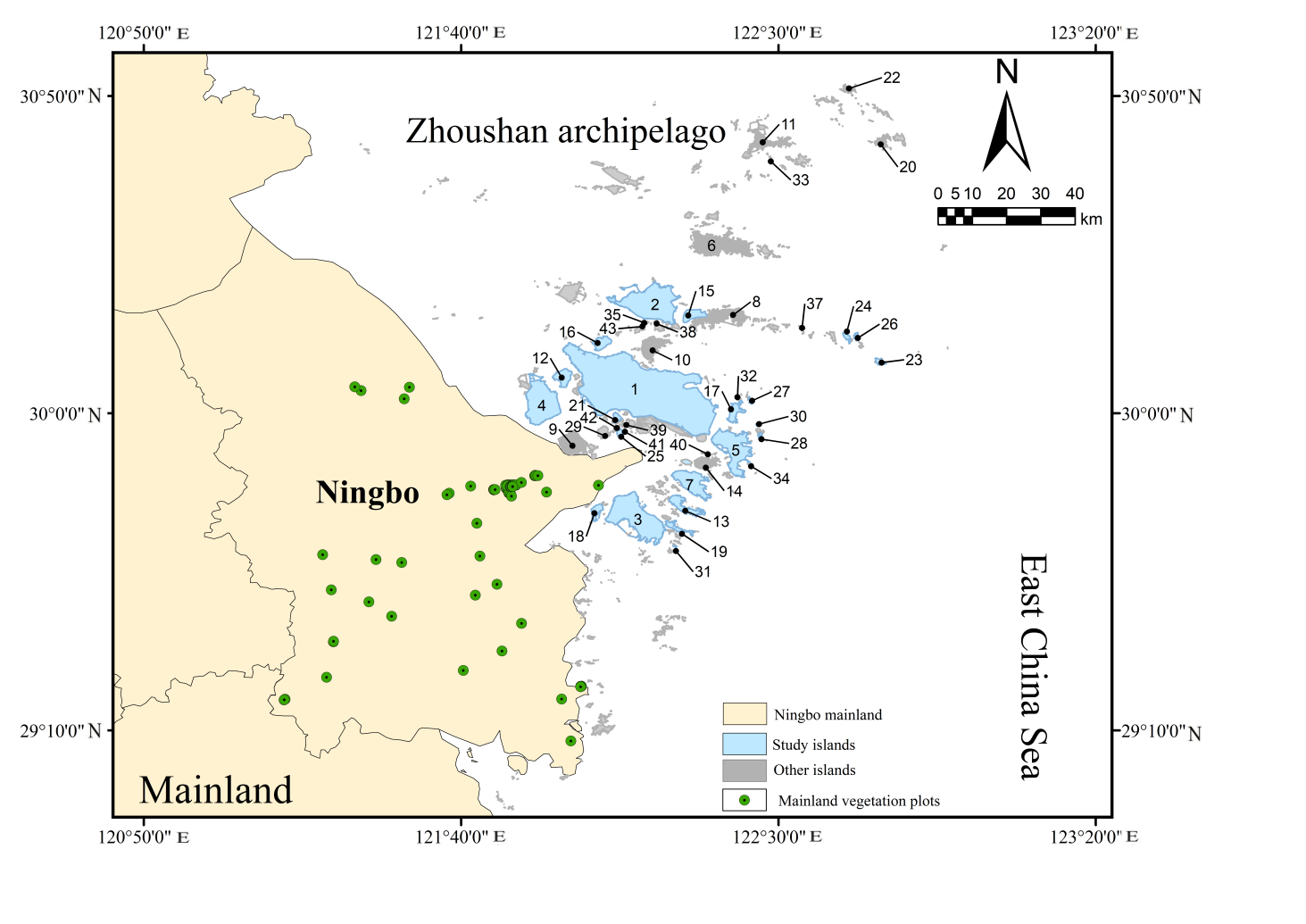
**

**Figure S1. Geographic locations of the Zhoushan archipelago, Ningbo mainland and research woody vegetation plots.**


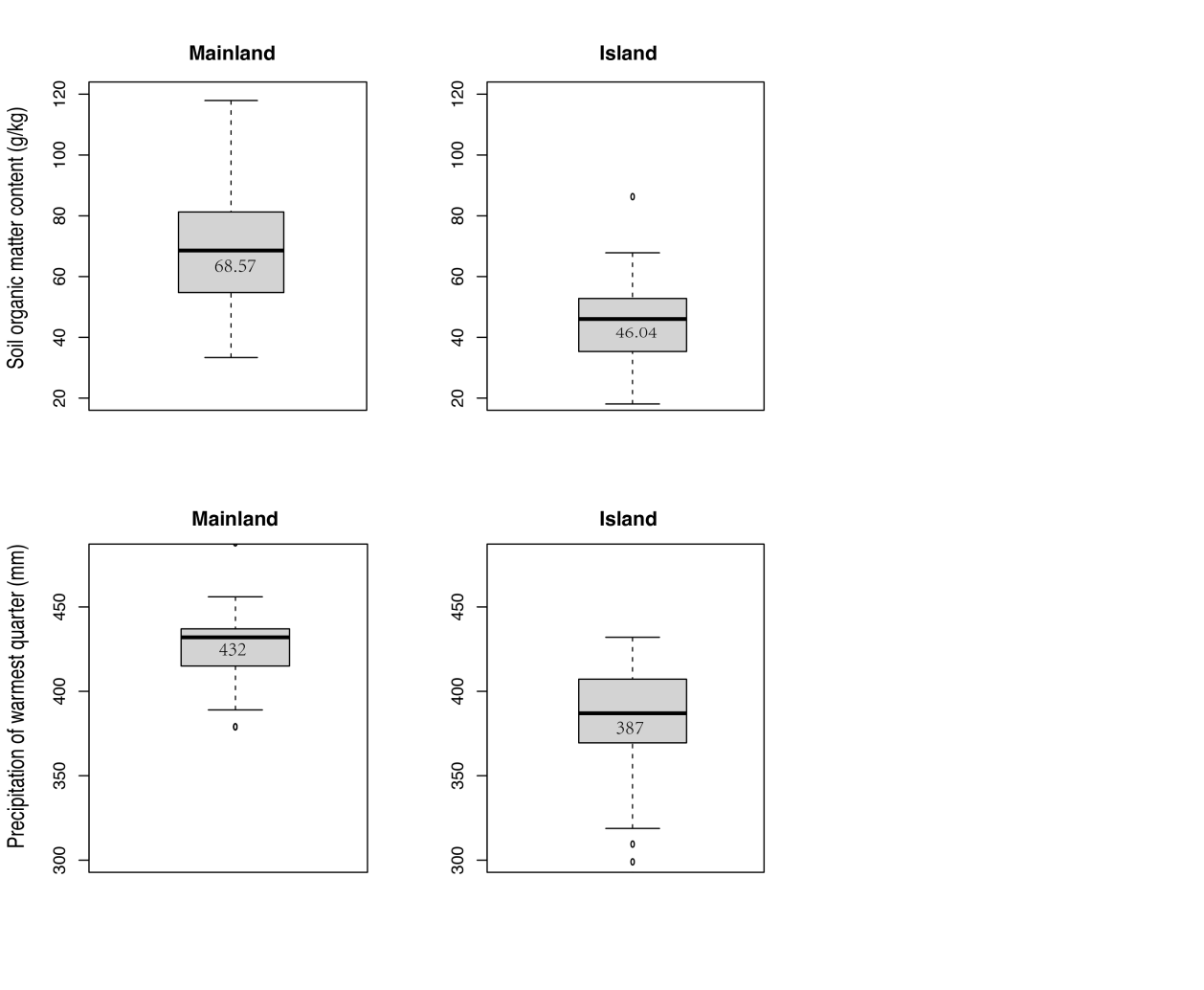


**Figure S2**. **Soil organic matter content and precipitation between mainland and islands.**

**Figure S3.** **The relationship between the 95th percentile of plant growth height and the 95th percentile of basal diameters.**

**Figure S4.** **The relationship between 5th percentile of the competitive index and 95th percentile of plant growth height.**


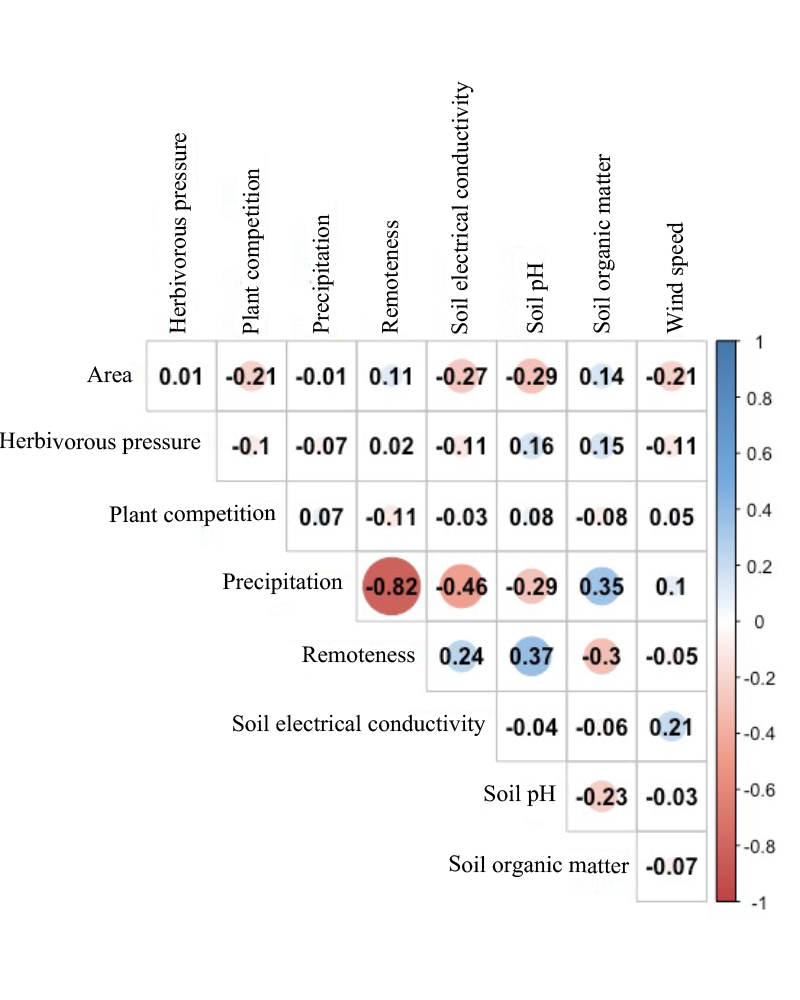


**Figure S5. Pearson's correlation matrix among island attributes, resource availability, environmental stress, relaxed competition and reduced herbivory, across 43 islands.** The value in the circle is the correlation coefficient.

**Figure S6.** **The effect of wind speed on the island rule-like patterns of plant size.**


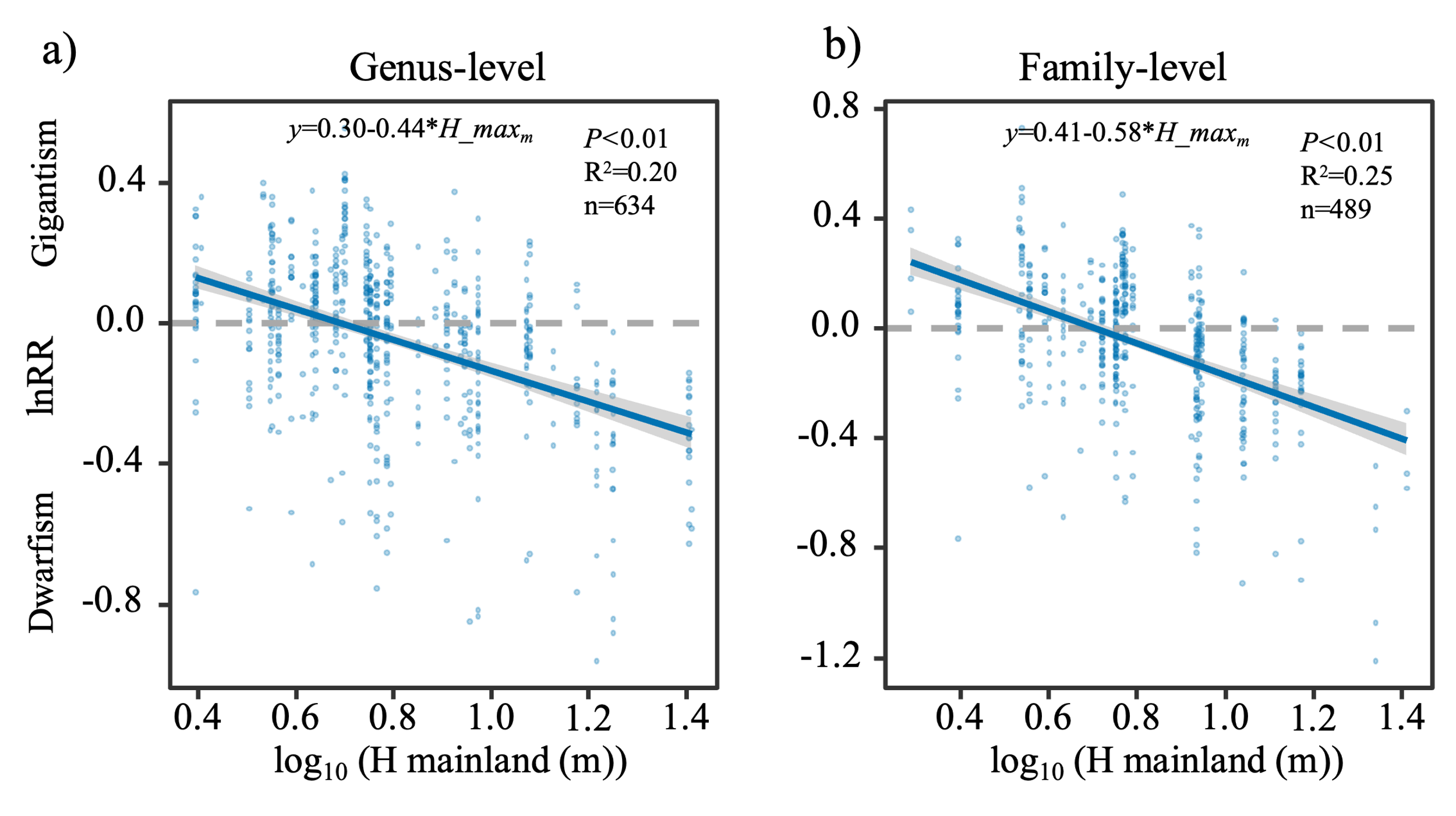


**Figure S7.** **The island rule-like patterns of plant size at (a) the genus level containing all 634 pairs, comparing the average height of genus on each island to the mainland and (b) the family level including 489 pairs, comparing the average height of family on each island to the mainland.** To test the plant size variation at the genus level, we first averaged the height of species within each genus on a given island and the mainland. Then, we compared the average height of genus on each island to that on the mainland. Similarly, to test the plant size variation at the family level, we averaged the height of species within each family on a given island and the mainland. Then, we compared the average height of family on each island to that on the mainland.
